# Risk Factors and 20-Year Time-Trend in Childhood Overweight and Obesity in Switzerland: A Repeated Cross-Sectional Study

**DOI:** 10.3390/children11091050

**Published:** 2024-08-28

**Authors:** Robin Berli, Chantal Sempach, Isabelle Herter-Aeberli

**Affiliations:** Laboratory of Nutrition and Metabolic Epigenetics, ETH Zurich, 8092 Zurich, Switzerland

**Keywords:** overweight, obesity, children, Switzerland, risk factors, diet, physical activity, media consumption, parental factors

## Abstract

**Background/Objective**: Even though global childhood obesity rates keep increasing, stabilization has been shown over the past decade in several countries, including Switzerland. We aimed to investigate the trends in childhood overweight and obesity over the past 21 years in Switzerland and to identify the associated risk factors. **Methods**: Using cluster sampling, we recruited a national sample of 6–12-year-old children in Switzerland (*n* = 1245). We conducted anthropometric measurements and assessed risk factors using a self-administered questionnaire. We investigated the time trend by including data from four comparable previous surveys conducted since 2002. **Results**: We found a prevalence of overweight, including obesity, of 16.1 (14.1–18.2)%, with a significantly higher proportion in boys (18.6 (15.5–21.6)%) compared to girls (13.7 (11.0–16.4)%). We found a small but significant reduction in the prevalence of overweight including obesity over time (*p* = 0.005), but not of obesity alone (*p* = 0.099). The most important risk factors for obesity were parental education, parental origin, media consumption, as well as several dietary factors. **Conclusions**: Despite a slight decreasing trend in childhood overweight in Switzerland, it remains a public health concern. Prevention programs should focus on migrant families and those with low education and emphasize the risks of sedentary behavior and the importance of a healthy diet.

## 1. Introduction

Globally, non-communicable diseases (NCDs) are one of the most important public health threats, leading to high morbidity and mortality rates and increasing socio-economic costs in each individual country [1]. Important behavioral patterns already developed during childhood have been shown to influence the NCD risk in adulthood [2,3,4,5]. By addressing and minimizing risk factors early in life, the development of NCDs such as obesity, cardiovascular disease (CVD), and diabetes can be delayed or even prevented in later life [6]. Childhood obesity is an important risk factor and was associated with strong evidence to NCDs in adulthood [2,4,5].

The global prevalence of childhood overweight and obesity keeps increasing [7]. Between 1971–1974 and 2017–2018, the prevalence of overweight including obesity (based on CDC references) in children and adolescents in the US increased from 16.4% to 41.5% [8]. Predictions further indicate a continued increase with 254 million children being obese by 2030 [7]. On the other hand, reports from a variety of countries since 2007 are pointing towards a stabilization in the childhood obesity prevalence, especially in high-income countries [9,10]. In Switzerland, we have seen a stabilizing trend of the obesity prevalence up to the year 2012 [11], and the last survey conducted in 2017/18 even showed a significant decrease, even though small, compared to the previous studies [12]. The latest Heath Behavior in School-Aged Children (HBSC) survey 2022 in Switzerland reported a prevalence of overweight including obesity comparable to that of 2018, except for 15-year-old girls, where an increase from 10.3%. to 12.2% was shown. Since no overall trend analysis between 2002 and 2022 was reported, it is, however, difficult to judge the general trend over time [13]. Furthermore, the WHO European Childhood Obesity Surveillance Initiative (COSI) has also reported a decrease or leveling off in the prevalence of overweight and obesity in several participating countries between round 4 (2015–2017) and round 5 (2018–2020), even though development did not go in the same direction for all countries [14]. Nevertheless, despite this evidence of a stabilization of the childhood overweight and obesity prevalence in parts of the world, global obesity rates are still projected to increase steadily at least until the year 2035, where 20% of boys and 18% of girls are expected to be obese (not including overweight) [15].

Several hypotheses have been developed to explain the stabilization of obesity in high-income countries, including the intervention hypothesis, stating a success of the promotion of more healthy dietary and physical activity habits, the saturation-equilibrium hypothesis, where any person pre-disposed to obesity in a certain environment will be obese, and the self-selection hypothesis, indicating the potential bias in recruitment of study participants based on the increased awareness of the problematic [9,10]. However, a systematic review has been unable to show a positive effect of policies promoting physical activity or healthy eating in adults so far [16]. In children, on the other hand, studies have associated a stabilization with changes in dietary intake (fruits and vegetables, sweetened beverages, solid margarines, and breakfast eating), as well as increased physical activity [17,18,19].

Despite the undisputable fact that ultimately a positive energy balance leads to weight gain, the underlying reasons responsible for the obesity epidemic are multifactorial. Changes in dietary patterns and physical activity levels can directly influence energy balance. But underlying factors such as socioeconomic background need to be taken into consideration. A systematic review has investigated the obesity prevalence trends related to the socioeconomic position in high-income countries, showing widening socioeconomic inequalities since 2000 [20].

In the current study, we aimed to assess the prevalence of overweight and obesity in a national sample of school-aged children in Switzerland and to determine the time trend in the prevalence since 2002. In addition, we aimed to assess risk factors for childhood overweight and obesity using a questionnaire.

## 2. Materials and Methods

### 2.1. Study Design

This study presents data of five national Swiss surveys. Results and methodology of the first four surveys conducted in 2002, 2007, 2012, and 2017/18 were reported previously [11,12,21,22]. For comparability reasons, the sampling strategy and measurement methods were kept the same. In brief, we used a probability proportionate to size cluster sampling based on current census data in order to receive a nationally representative sample of 6–12-year-old children in Switzerland. The country was divided into five regions, and in each region communities were stratified by size (<10,000 inhabitants, 10,000–100,000 inhabitants, and >100,000 inhabitants). Based on population size of each region and strata, the required number of schools was determined. In total, we aimed at recruiting 60 schools and ca. 45 children per school.

### 2.2. Enrollment and Participation

Participation in the study was voluntary both at the school and the individual level. Written informed consent was obtained from all parents and oral consent from the children in 2002, 2007, 2027/18, and 2023, while passive consent with the possibility for the parents to excuse their children from participation was applied in 2012. In all surveys, the first step of recruitment consisted in contacting randomly selected schools according to the sampling scheme. A school that refused or did not reply was systematically replaced by another school in the same strata until the intended sample size was reached or all available schools were contacted. Information letters for the parents and children were then sent to consenting schools for distribution among the children of the selected classrooms. Children were invited to participate when attending the selected classes, and aged 6–12 years. Approval for the previous studies was received by the responsible ethical committee as reported in the respective original publications [11,12,21,22]. The 2023 study was approved by the Cantonal Ethical Committee Zurich representing all other Cantonal Ethical Committees (BASEC-Nr. 2022-01713). Where necessary, cantonal or communal school or health authorities were asked for further authorization before contacting schools. The cantons Schaffhausen and Basel Stadt decided not to take part in the study. The study was registered on clinicaltrials.gov (NCT05697367).

### 2.3. Anthropometric Measurements

Identical measurement protocols for weight, height, waist circumference (WC), and skinfold thicknesses (SFT) were used in all studies, but WC measurements are lacking for 2002 and SFTs were not measured in 2007. A brief description including the equipment used in 2023 is given here. All measurements were performed by 5 trained investigators (CS, JS, LSP, GM, and RB). Children always left their classrooms in groups of 2–4 and measurements were conducted in a separate room with the children wearing light, indoor clothing. Body weight was measured to the nearest 0.1 kg using a digital balance (Soehnle, Style Sense Safe 100) and, using a transportable stadiometer (SECA, 213), height was measured to the nearest 0.1 cm. Using a non-stretchable measuring tape, waist circumference was measured midway between the lowest rib and the iliac crest.

SFTs measured at the biceps, triceps, subscapular, and suprailiacal were used to determine body fat percentage (BF%). We used a Harpender Skinfold Caliper with a resolution of 0.2 mm [23]. For the triceps, with the arm bent at 90 degrees, we marked the measurement point as the middle between the tip of the olecranon and acromial process. The SFT was then measured with the arm hanging freely, at the mark vertically above the olecranon. We used the same level to measure the SFT over the biceps, with the arm still hanging but the palm facing away from the body. For the subscapular measurement, we selected the site just beneath the lower angle of the scapula at 45° to the vertical following the natural cleavage lines of the skin. We measured the suprailiacal SFT above the iliac crest, slightly behind the midaxillary line and parallel to the cleavage lines of the skin, the arm gently held forward. We performed all measurements in duplicate on the right side of the body.

To determine inter-observer variability, measurements of WC and SFT were taken by two different investigators, while in the remaining participants, duplicate measurements were taken by the same investigator to assess intra-observer variability as the technical error of measurement (TEM) [24]:

$$TEM=\sqrt{(\Sigma D^{2}/2N}$$

where D is the difference between the two measurements and N the number of individuals measured. For better comparability between variables, relative TEM (%TEM) was then calculated as follows:

$$\%TEM=\left(\frac{TEM}{mean}\right)\times 100$$

### 2.4. Assessment of Risk Factors for Obesity

In the current survey, we distributed questionnaires to all participating children to assess factors related to physical activity, nutritional habits, socioeconomic background, and general health. We provided the questionnaire in the three main national languages, German, French, and Italian.

The questionnaire recorded the place of birth of the child and both parents. For the analysis, we combined the place of birth of both parents as follows: ‘Both Swiss’, ’Swiss and non-Swiss’, and ‘Both non-Swiss’. We further assessed the level of education of both parents as follows: ‘obligatory school time’, ‘apprenticeship without professional maturity’, apprenticeship with professional maturity’, ‘university of applied sciences or technical university’, ‘university’, and ‘other’. Where ‘other’ was chosen and a description given, this was assigned to the best fitting category. We combined the level of education of both parents and grouped them as follows: ‘low’ (obligatory school time), ‘moderate (apprenticeship with or without professional maturity), and ‘high’ (university of applied sciences, technical university, or university). It needs to be mentioned that the ‘college of higher education’ was categorized as apprenticeship with professional maturity, therefore being counted as a moderate level of education.

To assess physical activity, we inquired for how many days in a typical week the child was physically active for at least 60 min. Possible answers were as follows: ‘≤1 day/week’, ‘2–3 days/week’ ‘4–5 days/week’, and ‘≥6 days/week’. Furthermore, we sought information on the amount of time children spend in front of a screen in their free time and used this value to determine overall media consumption. Media consumption was grouped into ‘≤1 h/day’, ‘>1 h and ≤2 h/day’, ‘>2 h and ≤3 h/day’, and ‘>3 h/day’.

We asked about the frequency of consumption of several food groups over the past four weeks: sugar-sweetened beverages (SSB), artificially sweetened beverages, fruit and vegetable juices, fruits, vegetables (including salad), milk and dairy products, and meat. We grouped the answers given as follows. For soft drinks (with sugar or artificially sweetened), milk and dairy products, as well as meat and fish: ‘≤1 day/week’, ‘2–6 days/week’, and daily; for fruits and vegetables: ‘<1 time/day’, ‘2–4 times/day’, and ‘≥5 times/day’. We further asked the children whether they normally eat breakfast on weekdays and weekends.

We used additional questions to determine the general health status of the children. We assessed sleep duration as the difference between the time children usually go to bed and get up in the morning, both on the weekend and during the week. Furthermore, we inquired about a number of diseases (diabetes, asthma, other chronic disease, hay fever, ADHS, shortsightedness, celiac disease, eating disorders) and whether they felt generally well or not so well (feeling very well, well, rather well, or not well). Finally, children had to judge their own weight status (weight perception: much too thin, a bit too thin, about right, a bit too heavy, much too heavy) and rate their life in general on a scale from 1 to 10 (life satisfaction). An additional question asked about wellbeing using the WHO 5 questionnaire.

### 2.5. Data and Statistical Analysis

We conducted data and statistical analysis using EXCEL (Microsoft Office, Microsoft Corporation, Redmond, WA, USA) and IBM SPSS Statistics 28 (IBM Company, Armonk, NY, USA). We used RedCap, a research electronic data capture tool hosted by D-HEST at ETH Zurich for data collection. For all anthropometric, data this was performed directly at the schools and for the questionnaires at a later time point upon receipt, using a standardized procedure.

We calculated body mass index (BMI) as weight divided by height^2^. To calculate the overweight and obesity prevalence in in our study sample we used the CDC BMI reference values using the cut offs of the 85th and the 95th percentiles [25]. An increased risk for metabolic disease was defined based on Swiss reference values for WC with the 90th percentile defined as a cut-off [26].

To calculate the body density and with this the BF%, we used the mean value of the repeated SFT measurements, using the subsequent equations [27]:

BF% = (562 − 4.2 × [Age (y) − 2])/D − (525 − 4.7 × [age (y) − 2])

where D = body density

For boys:

D (g/mL) = 1.169 − 0.0788 × log10 (sum of 4 SFT [mm])

For girls:

D (g/mL) = 1.2063 − 0.0999 × log10 (sum of 4 SFT [mm])

We determined overweight and obesity from those measurements with the help of Swiss reference values for BF%. Overweight was defined as the 85th percentile and obesity as the 95th [28].

We used a chi-square test succeeded by a z-test (to determine significant differences between individual values) to compare prevalence of overweight and obesity between sex. Similarly, to identify differences in the prevalence of overweight and obesity between regions and communities of different sizes we used the chi-square test followed by a z-test (including Bonferroni correction for multiple comparisons). We estimated the 95% confidence intervals for all prevalences by applying the Wilson procedure [29] described by Robert Newcombe [30]. To investigate the trend in the prevalence of overweight and obesity between 2002 and 2023, we conducted a binary logistic regression and applied survey year as a continuous variable.

To study associations between BMI category and risk factors, we used multinomial logistic regressions. Each individual risk factor was tested with the dependent variable being the BMI category and each of the risk factors individually as the independent variable. The following 11 risk factors were tested: parental origin, parental education, media consumption, days physically active, sleep duration, breakfast consumption, as well as the frequency of consumption of sugar sweetened beverages, artificially sweetened beverages, dairy products, fruits and vegetables, and meat. The models were corrected for multiple testing of 11 factors using the Bonferroni–Holm correction.

Since the study design of all surveys used a probability proportionate to size cluster sampling, all data analysis was conducted with unweighted data. However, as recruitment based on the pre-defined numbers of schools per cluster was not successful in 2023, an additional analysis weighted by region was carried out for this survey for comparison.

## 3. Results

### 3.1. Response Rate of the 2023 Survey

In total, we contacted 690 schools, and 33 of those consented to participate, which resulted in a response rate of 4.8%. We were thus unable to reach the expected number of 60 schools. We invited 2707 children in the consenting schools to participate in the study and 1289 consented. Finally, 36 children were absent on the day of measurement, leaving a sample size of 1253, which represents 47.6% of the invited children. After excluding data of eight children aged ≥13 years, the number of children included for data analysis was 1245, corresponding to 0.23% of children in this age group in Switzerland. Relatively speaking, there was an underrepresentation of children from the central and eastern region (17% instead of 22%) and the north central region (15% instead of 18%), while the remaining three regions (western region (27% instead of 25%), northeastern region (34% instead of 30%), and southern region (8% instead of 4%)) were overrepresented. In total, we were only able to recruit ca. 50% of the intended sample size, which makes the current sample considerably smaller compared to the previous four surveys.

### 3.2. Inter- and Intra-Observer Variability in 2023

For the four SFT measurements, the calculated %TEM for inter-observer variability ranged from 3.7% (triceps) to 5.4% (suprailiacal) while it was 1.5% for WC. The inter-observer %TEM, on the other hand, ranged from 6.3% (triceps) to 12.9% (biceps) for the four SFTs and was 3.0% for WC.

### 3.3. Trends in Overweight and Obesity Prevalence between 2002 and 2023

**Figure 1:**
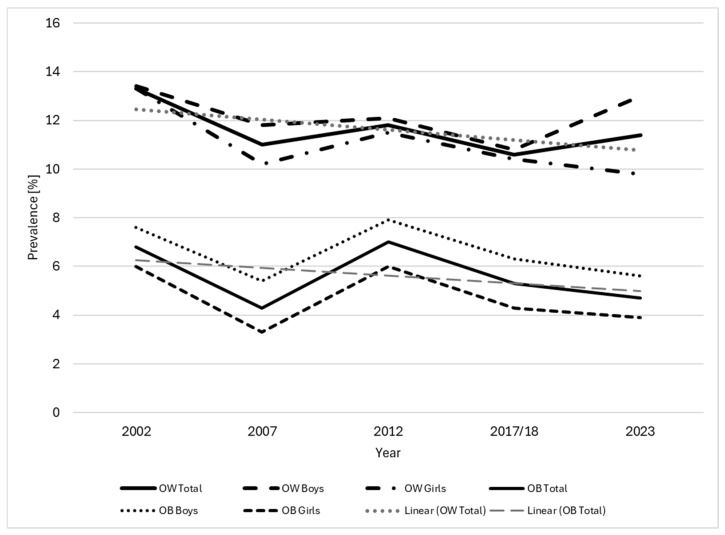
Trends in the prevalence of overweight (≥85th and <95th BMI for age percentile based on the CDC references) and obesity (≥95th BMI for age percentile) in children aged 6–12 years in national surveys between 2002 and 2023.

An overview of participant characteristics of all five included surveys is given in Table 1. To show the trend in the development of the prevalence of overweight and obesity data from all years is shown in Table 2 and Figure 1 for the total population as well as separated by sex.

We show significantly higher prevalence of overweight including obesity in boys compared to girls in 2007 and 2023 (*p* < 0.05), while the prevalence of obesity was significantly higher in boys compared to girls in the years 2007, 2012, and 2017/18 (*p* < 0.05) (compare Table 2). Overweight alone did not differ significantly by sex in any of the surveys.

As described above, we were unable to stick to the predefined sampling scheme to receive a sample proportionate to the population size of the different regions. In order to account for this in the data analysis, we have conducted an additional analysis of the prevalence in the 2023 survey using data weighted by region (Supplementary Table S1). Based on this analysis, unweighted data analysis may overestimate the overweight and obesity prevalence by 0.3% each compared to the weighted analysis, but generally led to comparable results. Therefore, all further results were calculated using unweighted data for comparability with the previous surveys.

We showed a slight but significant trend towards a lower prevalence of childhood overweight including obesity using a binary logistic regression (B(SE) = −0.011 (0.004), *p* = 0.005, OR = 0.989 (0.982–0.997)). On the other hand, we did not identify a significant reduction in the prevalence of obesity alone (B(SE) = −0.010 (0.006), *p* = 0.099, OR = 0.990 (0.978–1.002)). Divided by sex, we identified a significant decrease in overweight including obesity in girls (B(SE) = −0.014 (0.005), *p* = 0.008, OR = 0.986 (0.975–0.996)), but not in boys (B(SE) = −0.008 (0.005), *p* = 0.142, OR = 0.992 (0.982–1.003). For obesity alone, we did not find a significant reduction for either sex (*p* = 0.173 for girls and *p* = 0.290 for boys). Since the sampling procedure was slightly different in the year 2012 (passive consent) compared to all other surveys (active consent) an additional trend analysis on the development of overweight including obesity excluding this time point was carried out: B(SE) = −0.012 (0.004), *p* = 0.002, OR = 0.988 (0.981–0.996).

### 3.4. Risk Factors for Overweight and Obesity

Comparing the five regions of Switzerland, the prevalence (95% CI) of overweight ranged from 9.1% (5.2–13.0) in the central and eastern region to 17.8% (10.4–25.3) in the southern region and the prevalence of obesity from 2.2% (0.1–4.4) in the northcentral region to 9.9% (3.3–7.7) in the southern region, with no statistically significant differences. Considering community size, the prevalence of overweight was lowest in medium-sized communities (8.5% (3.4–13.5)) and highest in small communities (11.8% (9.7–14.0)), with large communities just behind (11.3% (7.4–15.2)). The prevalence of obesity was lowest in small communities (4.0% (2.7–5.3)), followed by medium-sized communities (4.2% (0.6–7.9)) and large communities (7.7% (4.4–11.0)). However, none of those differences were statistically significant.

In order to better understand potential risk factors associated with childhood overweight and obesity, we assessed a variety of potential risk factors using a questionnaire as described above. We received a completed questionnaire from 1126 children (90.4%). In the majority of cases, children completed the questionnaire with their parents (88.8%), while 8.4% of the children completed the questionnaire alone and in 2.8% of the cases, parents completed the questionnaire.

In the individual logistic regression models of risk factors on the prevalence of overweight and obesity, parental origin, parental education, media consumption, and the frequency of the consumption of sugar sweetened beverages, artificially sweetened beverages, fruits and vegetables, and dairy products showed a significant likelihood ratio test (*p* < 0.05). All those factors remained significant after correcting for multiple comparisons using Bonferroni–Holm correction. The detailed results of the univariate logistic regressions of these factors are shown in Table 3. We found no significant associations for the following factors: physical activity, eating breakfast, sleep duration, and the frequency of meat consumption. We found the highest OR for the risk of obesity in children of parents with a low education level (OR = 15.5 (5.6–42.7)), followed by the risk of obesity in children with both parents of non-Swiss origin (OR = 6.1 (3.1–12.1)). Only media consumption time seemed to affect the risk for overweight more strongly compared to that for obesity. In all other factors the risk for obesity was more strongly affected.

The questions related to general health perception and life satisfaction were analyzed in a descriptive way only. On a scale of 10 (best life you can imagine) to 1 (worst life you can imagine), normal weight and overweight children rated their life satisfaction with a median of 9, while the median was 8.5 for the obese group. Of the normal weight children, 82.4% selected a score of 8 or more, compared to 80.3% in the overweight and 65.2% in the obese group. When asked to judge their overall health status (from 1 for very good to 4 for bad), no child judged their health status as bad. The majority of children (65%) chose very good. In the different weight status groups, however, there were some differences: normal weight 66.7%, overweight 62.5%, and obese 35.6%. Nevertheless, 55.6% of the obese children still rated their overall health status as good and only 8.9% as just reasonably good. In the normal weight and overweight categories, 31.6% and 33.3% rated their health status as good and 1.7% and 4.2% as reasonably good. Overall, 76.1% of all children thought their weight was about right; in normal weight children, this applied to 78.8%, in overweight to 70.3%, and in obese to 37% of the children. On the other hand, 54.3% of obese children thought they were a bit heavy as compared to 26.3% of the overweight children and 5.7% of the normal weight children. In this last group, 13.8% thought they were a little too light.

## 4. Discussion

In this time-series study, we have for the fifth time assessed the prevalence of childhood overweight and obesity in a national sample of school-aged children living in Switzerland. Following the trend observed in the 2017/18 study [12], we confirmed a continued slight but significant reduction in the prevalence of overweight including obesity. Furthermore, also confirming previous findings, we identified parental education and parental origin as two of the most important predictors for obesity. However, in the current survey they were completed by media consumption as a proxy for sedentary behavior rather than physical activity as in the last survey. Furthermore, this is the first time that we identified several dietary factor as important risk factors.

### 4.1. Trends in Overweight and Obesity Prevalence between 2002 and 2023

After showing a stabilization of childhood obesity in 2012 [11] followed by a slightly decreasing trend in 2017/18 [12], our latest results confirm this trend to be a sustained development. Globally, childhood overweight and obesity are still increasing [31] and are projected to continue rising until 2030 at least [32]. Nevertheless, especially high-income countries have shown similar trends as we do in our study. The WHO European Childhood Obesity Surveillance Initiative (COSI) has reported a decrease or stabilization in several of the participating countries between round 4 (2015–17) and round 5 (2018–20) [14]. Similar trends were already reported in 2011 for nine countries throughout the world [10]. Nevertheless, trends may not always be sustained or not seen in all population groups in a similar way. In the US, for examples, data from the National Health and Nutrition Examination Survey (NHANEs) showed an increase in the prevalence of obesity between 2011/12 and 2017–20 [33], even though they had previously reported a stabilization between 2000 and 2007 [10]. In recent years, the COVID-19 pandemic may have further slowed down the efforts to combat childhood obesity as shown in the UK, where a steep increase in the prevalence of overweight and obesity was registered in 2020–21 [34]. In Switzerland, our study is not the only one reporting a stabilizing trend. The Health Behavior in School Aged Children (HBSC) survey conducted in 2022 also reported an overweight (including obesity) prevalence comparable to 2018 [13]. Furthermore, the BMI monitoring conducted by Health Promotion Switzerland in the cities of Zurich, Bern, and Basel also reported a decrease in the prevalence of overweight and obesity in the age group investigated here [35]. These findings of a sustained decrease in the prevalence of childhood overweight in Switzerland are promising. At the same time, it needs to be considered that the decrease is only weak and, if sustained this way, will not lead to major changes in the coming years.

### 4.2. Risk Factors for Overweight and Obesity

The questionnaire used to assess dietary, lifestyle, and socioeconomic factors associated with childhood overweight and obesity was similar to the ones administered in the 2012 and 2017/18 surveys [12,36], which allows comparison of the results. Comparable to those previous surveys, parental origin and parental education were again identified as important risk factors for overweight and particularly obesity. With an almost five- and seven-fold increased risk, respectively, children with two non-Swiss parents and with parents with a low education level are particularly disadvantaged. Since low education was also strongly correlated with non-Swiss origin, it can be expected, that this may be the main driver of the increased obesity risk rather than genetic background or cultural differences. An inverse association between parental education and the overweight and obesity prevalence of children has been previously shown in high income countries. A recent analysis using data from 24 countries included in the WHO European Childhood Obesity Surveillance Initiative (COSI) clearly showed this association in all high-income countries included, while an opposite trend was seen in upper-middle- and lower-middle-income countries [37]. Similarly, a large Spanish study showed more unfavorable developments in childhood obesity prevalence in children from the most deprived areas [38].

As opposed to our two previous surveys [12,36], we did not see a significant association between physical activity, assessed as the number of days a child was physically active for at least 60 min in a typical week, and the OR for overweight or obesity. Nevertheless, the OR for overweight was elevated in children who spent more time in front of a screen (media consumption time). Interestingly, a previous Swiss study already showed a negative relationship between time spent watching TV and parental education, setting up a link between these two factors [39]. The same study also identified an association between TV viewing time and the prevalence of overweight and obesity, similar to our results. Along the same lines, in the fourth round of COSI (2015–2017) screen time was higher in children from lower socioeconomic background and in the fifth round (2018–2020), lower parental education was associated with higher screen time [14]. In addition, increased media consumption has been shown to increase energy intake through snacking of energy-dense foods in front of the screen [39,40]. Even though there seems to be a certain discrepancy between the current survey and the two previous ones when it comes to physical activity and media consumption, there may be a simple explanation: if a child spends more time in front of a screen, there is less time for physical activity. Since physical activity was assessed using a questionnaire only and no tracking device or similar objective measurement, reporting errors related to misconception of the actual physical activity may have influenced the result. Especially young children are physically active throughout the day and not only when doing actual sports, thus the estimation of the duration can be much more challenging than estimating screen time.

None of our previous surveys could identify any dietary factors directly linked to the risk for obesity. Nevertheless, that diet has an effect on the development of obesity is probably undisputed. However, which dietary components in which quantities are problematic or beneficial is less clear. Our diet is composed of a variety of different foods that influence each other, thus ultimately the combinations and proportions will be just as important as individual foods. Yet, several foods and food groups have been associated with higher or lower levels of overweight or obesity in the literature. By constituting a large portion of the daily energy, one important factor repeatedly associated with overweight and obesity are sugar-sweetened beverages but sometimes also artificially sweetened beverages [41,42,43]. Furthermore, high consumption of fruits and vegetables has long been shown to be associated with a lower BMI [44], but also with a protective effect against the development of cancer and cardiovascular disease [45,46]. In agreement with this, we identified the frequency of consumption of fruits and vegetables, as well as that of sugar- and artificially sweetened beverages to potentially impact the OR especially for obesity. A healthy and balanced diet should therefore certainly continue to factor in any prevention programs or initiatives, but rather than focusing on one or two specific factors, the overall dietary composition should be emphasized.

### 4.3. Strengths and Limitations

Despite its strengths, our study also has limitations. We used the probability-proportionate to size cluster sampling with the aim to select a representative sample of school aged children in Switzerland, in order to have comparable data to the previous surveys. Due to the low response rate of the schools, however, it was not possible to complete the intended cluster sampling, which may have resulted in a less representative sample. The low response rate both in schools but also children of selected schools has further led to an overall sample size of only ca. 50% of what we aimed for. These two facts may have introduced a certain selection bias between consenting and not consenting schools as well as children. However, in order to avoid this selection bias as much as possible, the participant information did not focus on weight status at all, but on health and nutrition. Nevertheless, since anthropometric measurements were performed, some children or parents may have refused participation for fear of stigmatization. To control for a potential bias introduced by the sampling not being representative proportionate to the population size in the current survey, we have re-analyzed the prevalence of overweight and obesity weighting the data by region. The prevalence estimates that resulted from this weighted analysis, however, only differed minimally from the unweighted analysis. We therefore conclude that the unbalanced sampling based on the pre-defined clusters has not significantly influenced the study outcome.

In the current as well as all previous surveys, our investigators visited all participating schools in person and took anthropometric measurements that are comparable between schools. The combination of the administered questionnaire with the anthropometric measurements allows for a better insight into the effect of potential risk factors for the development of overweight and obesity. The questionnaire to assess risk factors was self-administered. Even though we requested children to complete the questionnaire at home seeking the help of their parents, this was only reported to have been the case in just below 90% of the questionnaires. Furthermore, estimating habitual physical activity or media consumption but also food intake is challenging and may have led to over- or underestimation.

## 5. Conclusions

To conclude, we have shown that over the past 21 years the prevalence of overweight including obesity, but not that of obesity alone, has decreased slightly but significantly in 6- to 12-year-old children in Switzerland. Despite this positive trend, with a total prevalence of 16%, childhood overweight and obesity remains an important public health issue. The most important predictors for the development of overweight or obesity were parental origin and education, with media consumption and several dietary factors also making a significant, but less consistent, contribution. Furthermore, we have again shown that overweight is more prevalent in boys compared to girls. Based on our findings, obesity prevention should continue to focus on specific groups of the population, namely those with a migration background and/or lower education levels and especially address boys. A further aspect to tackle could be sedentary behavior and nutrition education should be strengthened already at an early age in order for the children to develop healthier eating patterns.

## Figures and Tables

**Table 1:** Participant characteristics of five national surveys conducted in the years 2002, 2007, 2012, 2017/18, and 2023.

	2002	2007	2012	2017/18	2023
*n*	2493	2218	2963	2279	1245
Sex (% boys)	49.4	48.8	50.6	50.2	50.2
Age (y)	9.9 (6.2–13.0) ^a^	10.1 (6.3–13.0)	9.9 (6.3–13.0)	9.5 (6.0–12.9)	9.8 (6.1–12.9)
Weight (kg)	32.7 (17.7–94.4)	33.2 (15.9–83.3)	32.7 (16.7–132.3)	33.1 (16.7–106.2)	32.7 (15.4–78.7)
Height (m)	1.387 ± 0.120 ^b^	1.400 ± 0.116	1.389 ± 0.117	1.376 ± 0.111	1.393 ± 0.092
BMI (kg/m^2^)	17.1 (12.5–35.0)	16.9 (12.3–34.7)	16.9 (12.4–42.7)	17.2 (11.9–42.5)	16.8 (11.6–33.0)
Waist circumference (cm)	-	64.0 ± 8.0	63.2 ± 9.0	59.7 ± 7.1	61.7 ± 7.9
Body fat (%)	18.2 ± 9.0	-	19.3 ± 9.5	17.1 ± 8.0	16.5 ± 7.7
Nr. schools	57	60	58	60	38
Response rate children (%)	76.4	72.5	94.5	55.0	47.6

^a^ Median (min-max) (all such values), ^b^ Mean ± SD (all such values). BMI: body mass index, *n*: number.

**Table 2:** Prevalence (% (95% CI)) of overweight and obesity (based on CDC reference values) of five national studies in Switzerland between 2002 and 2023.

	2002	2007	2012	2017/18	2023
**Overweight incl. obesity**					
Total	20.3 (18.6–21.7) ^a^	15.3 (13.8–16.8) ^b^	18.8 (17.4–20.3) ^a^	15.9 (14.4–17.4) ^b^	16.1 (14.1–18.2) ^b^
Boys	21.2 (18.8–23.4) ^a^	17.2 (15.1–19.6) ^b,c^	20.0 (18.1–22.1) ^a,c^	17.1 (15.1–19.4) ^b^	18.6 (15.5–21.6) ^a,b,c^
Girls	19.5 (17.2–21.5) ^a^	13.5 (11.6–15.6) ^b,^*	17.5 (15.6–19.5) ^a,c^	14.7 (12.8–16.9) ^b,c^	13.7 (11.0–16.4) ^b,^*
**Overweight**					
Total	13.4 (12.1–14.8) ^a^	11.0 (9.8–12.4) ^b^	11.8 (10.7–13.0) ^a,b^	11.1 (9.8–12.4) ^b^	11.4 (9.6–13.2) ^a,b^
Boys	13.4 (11.4–15.5) ^a^	11.8 (10.0–13.9) ^a,b^	12.1 (10.6–13.9) ^a,b^	11.4 (9.5–13.3) ^b^	13.0 (10.3–15.6) ^a,b^
Girls	13.4 (11.6–15.2) ^a^	10.2 (8.6–12.1) ^b^	11.5 (10.0–13.2) ^a,b^	10.8 (9.2–12.7) ^b^	9.8 (7.5–12.2) ^b^
**Obesity**					
Total	6.9 (6.0–8.0) ^a^	4.3 (3.5–5.2) ^b^	7.0 (6.1–8.0) ^a^	5.4 (4.4–6.3) ^b^	4.7 (3.6–5.9) ^b^
Boys	7.8 (6.3–9.3) ^a^	5.4 (4.2–6.9) ^b^	7.9 (6.7–9.4) ^a^	6.3 (4.9–7.7) ^a,b^	5.6 (3.8–7.4) ^a,b^
Girls	6.1 (4.7–7.4) ^a,b^	3.3 (2.4–4.5) ^c,^*	6.0 (4.9–7.4) ^b,^*	4.4 (3.3–5.6) ^a,b,c,^*	3.9 (2.4–5.4) ^a,c^

* Significant differences between boys and girls for the respective year and weight category (chi-square test, *p* < 0.05). Different superscript letters (a, b, c) in rows indicate significant differences between survey years (chi-square test followed by z-test, *p* < 0.05). Overweight: >85th and <95th percentile, obesity >95th percentile.

**Table 3:** Univariate analysis of risk factors for overweight and obesity in a national sample of school aged children in Switzerland (*n* = 1126) analyzed using logistic regression (only factors that remained significant after controlling for multiple comparisons using Bonferroni–Holm correction are shown).

	Normal	Overweight			Obese		
	%	%	OR (95% CI)	*p*	%	OR (95% CI)	*p*
**Parental origin**							
Both CH	61.5	54.1	Ref.	-	29.2	Ref.	-
CH and non-CH	21.9	23.8	1.23 (0.78–1.96)	0.377	22.9	2.21 (0.99–4.93)	0.054
Both non-CH	16.6	22.1	1.52 (0.94–2.45)	0.090	47.9	6.09 (3.06–12.11)	<0.001
**Parental education**							
High	1.9	3.3	Ref.	-	17.5	Ref.	-
Medium	33.2	40.0	1.38 (0.93–2.04)	0.112	45.0	2.34 (1.16–4.71)	0.017
Low	64.8	56.7	1.96 (0.64–5.95)	0.236	37.5	15.53 (5.64–42.74)	<0.001
**Media consumption**							
<1 h per day	45.3	28.6	Ref.	-	31.9	Ref.	-
1–2 h per day	39.1	44.5	1.81 (1.15–2.84)	0.011	46.8	1.70 (0.87–3.32)	0.122
2–3 h per day	9.9	12.6	2.01 (1.05–3.84)	0.034	8.5	1.22 (0.39–3.75)	0.734
≥3 h per day	5.7	14.3	4.00 (2.09–7.65)	<0.001	6.9	3.20 (1.19–8.60)	0.021
**Sugar-sweetened beverages**							
≤1 per week	68.3	70.0	Ref.	-	41.7	Ref.	-
2–6 per week	21.8	20.0	0.90 (0.55–1.45)	0.654	29.2	2.20 (1.09–4.42)	0.028
≥1 per day	9.9	10.0	0.98 (0.52–1.87)	0.995	29.2	4.81 (2.35–9.85)	<0.001
**Artificially sweetened beverages**							
≤1 per week	93.7	90.0	Ref.	-	80.9	Ref.	-
2–6 per week	3.9	9.2	2.44 (1.21–4.92)	0.013	12.8	3.78 (1.50–9.49)	0.005
≥1 per day	2.4	0.8	0.36 (0.05–2.67)	0.315	6.4	3.04 (00.87–10.56)	0.081
**Fruits and vegetables**							
≥5 per day	12.9	8.2	Ref.	-	6.3	Ref.	-
2–4 per day	74.3	80.3	1.70 (0.86–3.34)	0.127	64.6	1.79 (0.54–5.94)	0.343
≤1 per day	12.9	11.5	1.40 (0.60–3.27)	0.438	29.2	4.67 (1.31–16.65)	0.018
**Dairy products**							
≥1 per day	66.2	62.0	Ref.	-	39.6	Ref.	-
2–6 per week	26.3	26.4	1.07 (0.69–1.66)	0.757	47.9	3.04 (1.63–5.68)	<0.001
≤1 per week	7.5	11.6	1.65 (0.89–3.07)	0.114	12.5	2.79 (1.08–7.22)	0.034

## Data Availability

The data presented in this study are available on request from the corresponding author in an anonymized form.

## Supplementary Materials

The following supporting information can be downloaded at: https://www.mdpi.com/article/10.3390/children11091050/s1, Table S1: Prevalence (%(*n*)) of underweight, overweight and obesity calculated using unweighted data and data weighted by region in a national survey in Switzerland conducted in 2023 (*n* = 1245).
