# Supplementary material for: Risk Factors and 20-Year Time-Trend in Childhood Overweight and Obesity in Switzerland: A Repeated Cross-Sectional Study
Source: Children (Basel). 2024 Aug 28;11(9):1050. doi: 10.3390/children11091050 (PMC11430791; doi:10.3390/children11091050)
Supplement: Supplementary file 1 [file children-11-01050-s001.zip › children-3148362-supplementary.pdf]

**Table S1:** Prevalence (%(n)) of underweight, overweight and obesity calculated using unweighted data and data weighted by region in a national survey in Switzerland conducted in 2023 (n=1245).

| | Underweight | Normal weight | Overweight | Obese |
|---|---|---|---|---|
| | **% (n)** | **% (n)** | **% (n)** | **% (n)** |
| **Unweighted data** | | | | |
| **Boys** | 3.5 (22) | 77.9 (487) | 13 (81) | 5.6 (35) |
| **Girls** | 4.5 (28) | 81.8 (507) | 9.8 (61) | 3.9 (24) |
| **Total** | 4.0 (50) | 79.8 (994) | 11.4 (142) | 4.7 (59) |
| **Weighted data** | | | | |
| **Boys** | 3.7 (23) | 78.7 (491) | 12.5 (78) | 5.1 (32) |
| **Girls** | 4.7 (29) | 81.9 (508) | 9.7 (60) | 3.7 (23) |
| **Total** | 4.2 (52) | 80.3 (999) | 11.1 (138) | 4.4 (55) |
